# CRISPR/Cas9 Genome Editing Introduction and Optimization in the Non-model Insect *Pyrrhocoris apterus*

**DOI:** 10.3389/fphys.2019.00891

**Published:** 2019-07-15

**Authors:** Joanna Kotwica-Rolinska, Lenka Chodakova, Daniela Chvalova, Lucie Kristofova, Iva Fenclova, Jan Provaznik, Maly Bertolutti, Bulah Chia-Hsiang Wu, David Dolezel

**Affiliations:** ^1^Laboratory of Molecular Chronobiology, Department of Molecular Biology and Genetics, Institute of Entomology, Biology Centre Czech Academy of Sciences, České Budějovice, Czechia; ^2^Department of Molecular Biology, Faculty of Sciences, University of South Bohemia, České Budějovice, Czechia

**Keywords:** CRISPR/Cas9, genome editing, non-model insect, genetic mosaicism, efficiency optimization

## Abstract

The CRISPR/Cas9 technique is widely used in experimentation with human cell lines as well as with other model systems, such as mice *Mus musculus*, zebrafish *Danio reiro*, and the fruit fly *Drosophila melanogaster*. However, publications describing the use of CRISPR/Cas9 for genome editing in non-model organisms, including non-model insects, are scarce. The introduction of this relatively new method presents many problems even for experienced researchers, especially with the lack of procedures to tackle issues concerning the efficiency of mutant generation. Here we present a protocol for efficient genome editing in the non-model insect species *Pyrrhocoris apterus*. We collected data from several independent trials that targeted several genes using the CRISPR/Cas9 system and determined that several crucial optimization steps led to a remarkably increased efficiency of mutant production. The main steps are as follows: the timing of embryo injection, the use of the heteroduplex mobility assay as a screening method, *in vivo* testing of sgRNA efficiency, and G_0_ germline mosaicism screening. The timing and the method of egg injections used here need to be optimized for other species, but other here-described optimization solutions can be applied immediately for genome editing in other insect species.

## Introduction

Non-model insect species have been used countless times to study many aspects of biology that are difficult to investigate using only *Drosophila.* Due to the lack of advanced molecular tools, most of the studies of gene function in non-model insects are based on the RNAi approach. Even though it was shown that RNAi could be efficiently applied for studying several processes, such as embryonic and postembryonic development (Niimi et al., 2005; Smykal et al., 2014; Schmitt-Engel et al., 2015; Sugahara et al., 2017), hormonal response pathways (Smykal et al., 2014; Sugahara et al., 2017), feeding (Meyering-Vos and Muller, 2007), the circadian clock (Moriyama et al., 2008; Kotwica et al., 2009; Uryu et al., 2013), and diapause (Sim and Denlinger, 2008; Ikeno et al., 2010; Kotwica-Rolinska et al., 2017), this approach has some limitations. The use of the RNAi leads to only partial knock-down of gene expression. The efficiency of the knock-down varies according to the type of tissues used, between different genes or among treated organisms. The usage of RNAi leads to off-target effects (Nandety et al., 2015) and is not inherited. Moreover, some groups of organisms, like butterflies, are shown to be highly resistant to the application of the exogenous dsRNA (Terenius et al., 2011). Therefore, there is a great need for developing advanced genetic tools, especially genome editing, for use in non-model insects.

CRISPR/Cas9 (Clustered Regularly Interspaced Palindromic Repeats/CRISPR-associated protein 9) is a cutting edge tool for relatively easy and efficient site-directed genome editing. This system consists of two main components: Cas9 RNA-guided nuclease and CRISPR RNA (crRNA) which targets Cas9 enzyme specifically to the sequence in the genome. This specificity is determined by a 20 nucleotide sequence complementary to the crRNA, which is followed by the PAM sequence (protospacer adjacent motif) - NGG. The activation of Cas9 nuclease also requires an additional RNA called *trans*-activating CRISPR RNA (tracrRNA), which binds to the crRNA forming single guide RNA (sgRNA). Cas9 nuclease activity leads to double-strand breaks in the target DNA sequences (Jinek et al., 2012) which are then repaired by cell machinery by the error-prone non-homologous end joining (NHEJ). These errors occurring during repair result in small insertions or deletions (so-called “indels”) or nucleotide substitutions, which eventually create a mutant version of the target gene. On the other hand, DNA breaks introduced by Cas9 can also be repaired by homologous recombination, with the use of modified donor template, allowing for introduction of various gene knock-ins (Ceasar et al., 2016; Sun et al., 2017; Thurtle-Schmidt and Lo, 2018).

The commercial availability of recombinant Cas9 enzymes, the low cost of guide RNA production, and the variety of online resources make genome editing an attractive, cost-effective, and straightforward tool even for small laboratories. Despite the significant advantages of the CRISPR/Cas9 system (Ceasar et al., 2016; Adli, 2018), only a limited amount of research has been done in non-model organisms, including non-model insects. To date, the CRISPR/Cas9 system has been applied in Diptera (Kistler et al., 2015; Meccariello et al., 2017; Choo et al., 2018; Sim et al., 2018), Coleoptera (Gilles et al., 2015), Hymenoptera (Kohno et al., 2016), Lepidoptera (Markert et al., 2016; Ye et al., 2017; Zhang and Reed, 2017), Hemiptera (Xue et al., 2018), and Orthoptera (Li et al., 2016), and the use of this system in non-model insects has also been reviewed by Sun et al. (2017). Immediate updates about new genetically engineered insects are available at the Insect Genetic Technologies Coordination Network (IGTRCN) website^1^.

Although there are protocols and online resources available for genome editing in various organisms such as fruit flies (Bassett and Liu, 2014; Housden et al., 2014; Port et al., 2014; Gratz et al., 2015; Bier et al., 2018), mosquitoes (Kistler et al., 2015), butterflies (Zhang and Reed, 2017; Banerjee and Monteiro, 2018), mice (Henao-Mejia et al., 2016), and humans (Lee et al., 2017), there is a great need to optimize the method when using it in a new model species. There are several different bottlenecks in optimizing CRISPR-Cas9 experiments, and numerous steps are crucial for an efficient mutant generation. Certain issues depend on the biology of the particular species, including the development of the method and specific timing of the embryo injection. Other issues are common for most organisms and include optimization of CRISPR/Cas9 system, such as design and prediction of guide RNA efficiency, testing the cutting efficiency *in vivo* and optimizing protocols for mutant screening.

While attempting to introduce CRISPR/Cas9 system in the non-model insect *Pyrrhocoris apterus* (Heteroptera, Pyrrhocorridae) we encountered several problems concerning efficient mutant generation. During many attempts of creating knockout mutants for several genes in *P. apterus*, we gradually increased the efficiency of CRISPR/Cas9. Optimization of several steps, resulted in significantly reduced cost and workload required for successful mutant production. This paper is a summary of many independent experiments and describes a complete and detailed workflow together with CRISPR/Cas9 method optimization for the efficient genome editing in non-model insect.

Our laboratory is focused on the circadian clock mechanism and seasonality in the non-model insect – the linden bug, *P. apterus* (Heteroptera, Pyrrhocorridae). Therefore, we focused on editing genes expected to be involved in the circadian clock machinery and we targeted: part of the *cryptochrome2* gene (Yuan et al., 2007; Bajgar et al., 2013) coding for the N-terminal and C*-*terminal protein regions, *timeless* gene (Vosshall et al., 1994), 3 regions of the *period* gene, known to differently affect the pace of *Drosophila* circadian clock (*per^S^, per^L^*, and *per*^SLIH^) (Konopka and Benzer, 1971; Hamblen et al., 1998), and *pigment dispersing factor* (*pdf*), a neuropeptide involved in the circadian clock output (Park and Hall, 1998; Renn et al., 1999). These genes are known to be non-essential for survival, development, or fertility in *Drosophila*, therefore, suitable for testing CRISPR/CAS technology in *P. apterus*. We also targeted one novel putative neuropeptide, *TEFLamide*, which function is unknown in insects.

## Materials and Methods

### Needle Preparations

Needles used for egg injections were made of borosilicate glass capillaries with a filament (outer diameter 1 mm, inner diameter 0.58 mm) (Sutter Instrument, Germany). Needles were pulled with the Magnetic Glass Microelectrode Horizontal Needle Puller PN-31 (Narishige, Japan) using the following settings: temperature 83°C, magnet sub 33 and magnet main 96. Just before injections, the tip of the needle was gently scratched with the fine forceps, allowing the needle to open with the tip angled at around 45°.

### Egg Injections

For our experiments, three different ways of egg injections were carried out. In the beginning, eggs were collected every 2 h and injected immediately [0–2 h after egg laying (AEL)]. For subsequent experiments, eggs were collected every 2 h, and injected 2 h afterward (2–4 h AEL). However, in most experiments, bugs were allowed to lay eggs for 12 h and then injections were immediately administered (0–12 h AEL).

**FIGURE 1:**
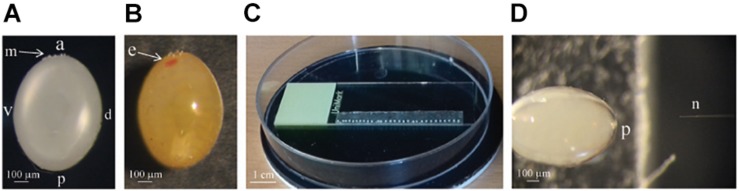
**(A)** A picture of the 4 h old *Pyrrhocoris apterus* egg, a = anterior, p = posterior, v = ventral, d = dorsal, m = ring of micropyle. **(B)** Picture of the egg on the sixth day of the development, developing eye (e) is clearly visible in the anterior part of the egg. **(C)** Picture of the eggs 0–12 h AEL lined up on the double-sided adhesive tape prepared for injection. **(D)** The size of the needle (n) compared to the size of the *P. apterus* egg. The posterior part (p) of the egg is being injected

Eggs were attached to the microscope glass slide using double-sided adhesive tape, with the posterior end directed to the edge of the glass slide (Figure 1C). Eggs were then kept in the Petri dish and covered with distilled water for 15 min. Afterward, injections were carried out under a stereoscopic microscope (Leica DSK 500) with eggs still completely submerged in water. Cas9 and guide RNA mixture was injected either into the middle of the egg (when 0–2 h AEL eggs were used) or into the posterior side of the egg, around the site where germ cells are generated (when older eggs were used) (Figure 1D).

Eggs were injected with the FemtoJet system (Eppendorf) with the following settings: injection pressure (Pi): 100–300 hPa, time of injection 0.3 s and compensation pressure (Pc) 30–60 hPa. The exact settings were adjusted for every needle separately. Later, injected eggs were delicately dried out with a paper-tissue and the hole made by the needle was covered with commercially available cyanoacrylate glue. Eggs in petri dishes were supplied with moist paper-tissues and transferred to the incubator where they were kept until hatching (LD18:6 26°C). After 8–9 days, hatched larvae were transferred to the new Petri dish supplied with water and linden seeds and allowed to grow until adulthood (G_0_ adults).

### Injection Mixture

#### Cas9 Source

Cas9 mRNA was produced by *in vitro* transcription as previously described (Kistler et al., 2015). Cas9 mRNA was transcribed from plasmid MLM3613 (Addgene) using the mMESSAGE mMACHINE T7 Ultra Transcription Kit (Thermo Fisher Scientific), which includes reagents for Poly(A) tailing. The quality of Cas9 mRNA was checked on the agarose gel, and the concentration was measured with the NanoDrop 2000 spectrophotometer (Thermo Fisher Scientific). The mRNA was then diluted to the concentration of 1 μg/μl and stored at −80°C until further use.

For most experiments commercially available lyophilized Cas9 protein (CP01 PNA Bio) was used. The protein powder was resuspended in the nuclease-free water at the room temperature for 10 min to the concentration of 1 μg/μl and stored at −80°C until further use. Cas9 mRNA was mixed with the sgRNA and injected immediately. In the case of Cas9 protein, to obtain ribonucleoprotein complexes, Cas9 was mixed with guide RNA and kept at the 25°C for 30 min prior injections.

#### sgRNA Source

Most of the single guide RNAs (sgRNAs) used in this study were prepared from crRNA artificially fused to the tracrRNA by the non-template PCR, followed by *in vitro* transcription (Kistler et al., 2015). Non-template PCR mixture was composed of primer pairs.

• CRISPR Reverse **(5 μl, 10μM)** primer, which is universal for all gRNAs:

5′AAAAGCACCGACTCGGTGCCACTTTTTCAAGTTGAT AACGGACTAGCCTTATTTTAACTTGCTATTTCTAGCTCTA AAAC 3′

CRISPR Forward primer **(5 μl, 10μM), which contains** T7 promoter sequence, 20nt long region specific for each gRNA (N20) and **two guanines (*GG*)**, which are necessary for *in vitro* transcription (therefore, GG are either present in the original target sequence or added)

5′GAAATTAATACGACTCACTATA ***GG*** (N20) GTTTTAGA GCTAGAAATAGC 3′

• KAPA HiFi Fidelity Buffer (5X) (**20** **μl**)
• KAPA dNTP Mix (10 mM each) (**3** **μl**)
• KAPA HiFi DNA Polymerase (**2** **μl**)
• Nuclease free water (**65 μl**)

PCR was run at following parameters: 3 min at 95°C, (98°C – 20 s, 58°C – 30 s, and 72°C – 15 s) for 35 cycles, followed by elongation at 72°C for 5 min.

Whole PCR product was run on the 1.5% agarose gel in TAE buffer. Bands were excised from the gel and column purified with the QIAquick Gel Extraction Kit (Qiagen), according to provider’s instructions. This additional purification step allowed for better removal of non-ligated primers, especially the forward primer containing a T7 promoter region, and thus, lowering the chance of production of truncated versions of the chimeric guide RNA. *In vitro* transcription was performed with MegaScript T7 Kit (Ambion). Reaction mix was composed of:

• 50–300 ng of the purified PCR template.
• 10x reaction buffer (2 μl)
• rATP, rUTP, rGTP, rCTP mix (2 μl)
• T7 enzyme mix (2 μL)
• nuclease free water to 20 μl

*In vitro* transcription was carried out for 2.5 h at 37°C. Afterward DNA template was degraded by the addition of add 1 μL of Turbo DNAse and 15 min incubation at 37°C. sgRNAs were then purified by 3M sodium acetate and ethanol precipitation. The quality of Cas9 mRNA was checked on the agarose gel, and the concentration was measured with the NanoDrop 2000 spectrophotometer (Thermo Fisher Scientific). Normally, in our hands, the yield of the sgRNAs was between 150 and 400 μg. sgRNAs were diluted to the concentration of 1 μg/μl, aliquoted and stored at −80°C until further use.

Embryos were injected with the various concentrations of the sgRNAs ranging between 200 and 500 ng/μl.

In addition to the *in vitro* transcribed guide RNA (PDF 1), a commercially produced crRNA (PDF 1 crRNA+tracrRNA) (Sigma) targeting the same sequence was ordered for *pigment dispersing factor* (*pdf*). crRNA and tracrRNA were injected into embryos in equimolar concentrations of 18 μM or 9 μM each (238 ng/μl and 400 ng/μl or 119 ng/μl and 200 ng/μl, respectively).

All combinations of different concentrations of the Cas9 mRNA or Cas9 protein and guide RNAs used in this study are described later in the text and are listed in the Supplementary Table 1.

### *In silico* Guide Design and Efficiency Prediction

All guide RNAs tested in this study (Table 1) were designed using open access online CRISPOR software^2^ with “no genome” option in step 2, and “20-bp – NGG for Sp Cas9” PAM motif in step 3. As a result CRISPOR software gives a table with all possible on-target sequences and also calculates efficiency of the guide RNA, which is predicted by different algorithms for guides produced by T7 *in vitro* transcription and for guides which transcription is driven by the U6 promoter.

**TABLE 1:** List of targeted genes and guide RNAs designed and tested in this study.

**Gene name**	**Targeted protein region**
	**(No. of guide RNAs tested)**
*cryptochrome 2* (*cry2*)	N-terminal (3)
	C-terminal (3)
*timeless* (*tim*)	Middle of the protein
	Exon 7 (4)
	Exon 8 (1)
*period* (*per*)	PER “short” region (2)
	PER “long” region (3)
	PER “SLIH” region (5)
*pigment dispersing factor* (*pdf*)	Active neuropeptide (4)
*TEFLamide* (*TEFL*)	Predicted active neuropeptide (9)

### *In vivo* sgRNA Efficiency Testing

Twenty-nine different sgRNAs targeting 5 genes (Table 1) were produced by *in vitro* transcription as described above. Each of the sgRNAs (200 ng/μl) was injected together with Cas9 protein (500 ng/μl) into approximately 30 eggs laid by bugs overnight (0–12 h AEL). After 30 h post-injection, eggs were collected and frozen at −20°C until further use. Eggs were squished by the pipette tip in 100 μl of the squishing buffer (10 mM Tris-HCl pH = 8.0, 1 mM EDTA, 25 mM NaCl, 200 μg/ml Proteinase K) and incubated for 1 h at 37°C. Proteinase K was then heat inactivated for 3 min at 95°C and 1 μl of homogenate was used for PCR reaction. The typical 10 μl PCR reaction consisted of 5 μl of 2x TP 2x Master Mix (TopBio, Czechia), 0.25 μl of each 10 μM specific primers and 3.5 μl of nuclease-free water. PCR was performed in a thermocycler with the following parameters: 3 min at 94°C, (94°C – 30 s, specific for each primer set annealing temperature – 30 s, and 72°C – 30 s) for 35 cycles, followed by elongation at 72°C for 10 min. All the primers used in this study are listed in the Supplementary Table 1.

### Heteroduplex Mobility Assay

Five microliters of the PCR reaction mixture was loaded onto 15% non-denaturing PAGE gels [15% acrylamide-bisacrylamide (29:1, w/w), 1X Tris-borate-EDTA (TBE), ammonium persulfate, and TEMED] and run in 0.5X TBE buffer for 2 h at 150 V in Mini PROTEAN Electrophoresis System (Bio-Rad), similar to the previously described protocol (Zhu et al., 2014). The gels were then stained for 10 min with GelRed nucleic acid stain solution in water (Biotium, United States) and imaged by Gel Documentation System Smart3-EZ (VWR, Belgium).

### Detection of Mosaicism in G_0_ Generation

For somatic mosaicism testing, one antenna of the adult G_0_ bug was cut out, and DNA was extracted by squishing the antenna with the pipette tip in 50 μl of the squishing buffer. PCR and heteroduplex mobility assay analysis was done as described above. An identical method was used later for a screening of heterozygotes in the F_1_ generation.

For germline mosaicism testing, G_0_ bugs were allowed to mate with wild type partners until F_1_ progeny appeared (around 2 weeks). Next, G_0_ generation bugs were sacrificed and, ovaries and testes were dissected out and stored at −20°C until further use (no longer than 1 month). After thawing, gonads were gently squished, but not homogenized, in 100 μl of the squishing buffer followed by DNA isolation, PCR and heteroduplex mobility assay analysis as described above.

### Biosafety

The work on the genetically modified *P. apterus* was carried out under approval from the Ministry of the Environment of the Czech Republic. All work was performed in the class 1 GMO approved laboratories and the personnel was trained to work according to biosafety procedures. To our knowledge none of the modifications should increase the selective advantage of the modified over naturally occurred bugs. No modified bug was released to the environment and all redundant bugs were sacrificed.

## Results and Discussion

Our laboratory is studying circadian clock, seasonality and physiology in *P. apterus* (Bajgar et al., 2013; Dolezel, 2015; Pivarciova et al., 2016; Urbanova et al., 2016; Kotwica-Rolinska et al., 2017). Because of the lack of available mutants, functional research in this species is limited to the RNAi technology. Therefore, in addition to the regular RNAi studies, we also implemented the site-directed gene editing by the CRISPR/Cas9. During our attempts we gradually increased the efficiency of mutant production by optimizing several steps including sgRNA efficiency testing, timing of embryo injections, and mutant screening methods. While the description of the phenotype of mutants will be provided elsewhere, in this paper, based on our collection of data from several independent experiments, we provide a complete protocol for efficient *P. apterus* gene knockout by CRISPR/Cas9. Moreover, in this paper, we discuss the problems encountered and the optimization steps implemented toward increased efficiency of the mutant generation.

### Injections

*Pyrrhocoris apterus* eggs are about 1 mm long, white, and non-transparent, with a clearly visible ring of micropyle at the anterior pole of the egg, slightly shifted toward the ventral side (Figures 1A,B). A *P. apterus* egg is covered with a very hard chorion and only harsh conditions, that is 10 min of treatment with 30% KOH followed by 7 min of treatment with 50% commercial bleach, can remove it. Since this approach kills the embryos, all injections in this study were performed on chorionated eggs. Two main characteristics of the *P. apterus* egg make administering injections challenging. First, the very hard chorion breaks needles very easily. Second, the high pressure inside the egg causes breaks in the chorion during injection followed by immediate death of the embryo. Additionally, high pressure inside the egg pushes the egg cytoplasm into the needle, clogging it completely and the leak of the cytoplasm also results in embryonic death. However, we found that immersing eggs into distilled water for 15 min before injection softens the chorion which in turn decreases the pressure inside the egg. When eggs are dried out, both the chorion strength and the internal egg pressure are restored immediately; therefore, all injections were performed when eggs were immersed in water. The injection procedure lasts for 1 h at most, and our test has shown that even 2 h of immersion in water does not decrease embryo survival (Table 2). After injections eggs were kept in Petri dishes with the moist paper towel to maintain high humidity, and we found this to be critical for embryo survival (Table 3) increasing it from 1% to almost 40%, when eggs were injected with nuclease free water only. The other treatment that was found to increase embryo survival by around 17% (when eggs were injected with water only) was covering the hole made by the needle with a small drop of commercially available acrylic glue (Table 3).

**TABLE 2:** Embryo survival after 0–2 h immersion in water.

**Time of immersion**		
**in water (min)**	**No of eggs**	**Hatched larva (%)**
0	50	68
30	50	62
60	50	78
90	50	72
120	50	78

**TABLE 3:** Embryo survival after water injection and different post-injection treatment.

**Treatment**	**No. of eggs**	**Hatched larva (%)**
Control	137	77.0
Water injection	80	1.2
Water injection + glue	73	2.7
Water injection + humid chamber	71	33.8
Water injection + humid chamber + glue	63	50.8

As a first target, the start of the coding region of the *cryptochrome 2* (*cry2*) gene was selected. Eggs were injected immediately after collection (0–2 AEL), with the Cas9 mRNA combined with one of the three different sgRNAs: N-cry2_2, N-cry2_3 or N-cry2_8 (Supplementary Figure 1). The hatchability of the injected eggs varied between 18.9 and 32.8 % and later, the survival until adulthood stood between 60 and 70% (Supplementary Table 1). G_o_ survivors were crossed with wild type bugs and allowed to mate until their death. Next, all the F_1_ were maintained until adulthood and then one antenna was cut and was subjected to DNA isolation, PCR, and agarose electrophoresis to check for the occurrence of heterozygosity in the target region. Based on this approach we detected six heterozygous mutants in F_1_ generation out of over 2000 F_1_ individuals screened (Supplementary Table 1). All of them were obtained with the injection of the sgRNA N-cry2 8 (Supplementary Figure 1).

Although this method of mutant generation was successful, the final number of mutants was very low when compared to the number of individuals screened. During later experiments, optimization of several steps allowed for higher success in the mutant generation and lowering cost, time and labor needed for their production.

### Screening Method

Most of the recently published research on CRISPR/Cas9 genome editing in non-model insects focuses on genes that show visible phenotypic effect (eye, or body color change or loss of the fluorescence) upon disruption (Gilles et al., 2015; Meccariello et al., 2017; Sun et al., 2017; Zhang and Reed, 2017; Choo et al., 2018; Sim et al., 2018; Xue et al., 2018). In our case, a mutation in none of the genes of interest shows noticeable phenotypic changes that can be used as a marker for screening. A genome-wide screening, mainly by the PCR amplification of the mutated region is needed to analyze the changes in the sequence associated with the phenotype. CRISPR Cas9 and NHEJ produce indels in a particular region, generally between the third and the fourth nucleotide upstream of the NGG sequence (Jinek et al., 2012). A similar effect was also observed in our mutant lines, where most of indels were located in close proximity of the predicted cut site (Supplementary Table 2). The reported indels produced by sgRNA can be as small as 1 bp and ranges up to hundred nucleotides (rarely), but most often, as we also observed, detected indels are within the range of −/+ 10 nucleotides (Supplementary Table 3). In case of big indels, traditional PCR and agarose electrophoresis are sufficient to identify heterozygotes easily. However, with this method, the smaller the change, and the harder it is to see the difference between wild type and mutated allele (Supplementary Figure 1C). There are many other screening techniques, which differ in sensitivity, cost, and the labor and equipment needed (Bassett and Liu, 2014; Zhu et al., 2014; Kistler et al., 2015; Zhang and Reed, 2017; Zischewski et al., 2017; Banerjee and Monteiro, 2018). Among the available methods of screening, we used the heteroduplex mobility assay (Zhu et al., 2014). In short, in heterozygotes, heteroduplex DNA formed by one strand of WT DNA and one strand of DNA with an indel will migrate slower than homoduplex DNA, when using polyacrylamide gel electrophoresis (PAGE). Supplementary Figure 1C shows a comparison between the resolution of the 4% agarose and 15% polyacrylamide gel in the detection of *cry2* heterozygotes with −5 bp, +4 bp, +13 bp, and +27 bp indels. It has to be taken into consideration that for a better resolution in the heteroduplex mobility assay, primers used should show high specificity, and the PCR product length should be around 100 bp. The bigger the PCR product, the lower the resolution is obtained.

### Cas9 Source

There are three primary sources of Cas9 delivery used for injections in genome editing experiments: (a) expression plasmid producing Cas9 mRNA and translated to protein in the host cell, (b) mRNA of Cas9, and (c) Cas9 protein (Bassett and Liu, 2014; Housden et al., 2014; Gratz et al., 2015; Kistler et al., 2015; Thurtle-Schmidt and Lo, 2018). Expression plasmids producing a high level of Cas9 protein are widely used for *Drosophila* studies. The most common promoters used are *Actin-5c*, *nanos*, *tubulin*, or *hsp70* promoter^3^. Although success has been shown with *actin* promoter induced luciferase expression in mosquitoes (Zhao and Eggleston, 1999) and activation of heterologous gene expression system in cell lines of different species of origin (fly and mammals) (Chavez et al., 2016), there is not enough data showing that this system will work efficiently in other species as well. Therefore, choosing and testing the right expression vector will cost time and money. Furthermore, the stability of the expression vector and constitutive production of Cas9 protein can increase off-target cuts in the genome (Zhang et al., 2015).

For our studies, two approaches were compared for genome editing efficiency – (i) injection of the Cas9 mRNA produced by *in vitro* transcription, and (ii) use of the available Cas9 protein. The efficiency of mutant production by Cas9 mRNA and Cas9 protein (two different concentration were used) was compared in 3 different genes: *period* (short region 2 different guides were used perS 1 and perS 4), *pdf* (PDF 1), and *TEFLamide* (TEFL 2).

Similar to the studies described by Kistler et al. (2015), better results were obtained while using recombinant Cas9 protein compared to Cas9 mRNA for injections (Table 4) (*p* < 0.05, Welch’s *t*-test). When Cas9 mRNA was injected into G_0_ eggs, we did not observe F_1_ heterozygotes in any of the tested genes. In contrast, when Cas9 mRNA was replaced by the Cas9 protein in the injection mixture, we observed the occurrence of 2 (perS 1), 1 (perS 4), 1 (PDF 1) and 2 (TEFL 2) heterozygotes in the F_1_ generation. There was no difference in the number of heterozygotes in the F_1_ generation (*p* > 0.05, Welch’s *t*-test) (Table 4) or bugs survival (Supplementary Table 1) when two different concentrations (250 ng/μl or 500 ng/μl) of Cas9 protein were injected. Therefore, further experiments were performed with a higher concentration of the Cas9 protein (500 ng/μl) in the injection mixture. Increased efficiency with the use of the Cas9 protein in the mutant generation is most probably caused by the higher stability in the host cells. Even though the Cas9 protein is more expensive than the Cas9 mRNA production by *in vitro* transcription, the efficiency of the mutant production favors the Cas9 protein as the preferred choice of this approach. However, the switch from using Cas9 protein in place of the Cas9 mRNA did not significantly increase the efficiency of mutant generation. The final efficiency of the genome editing was still unsatisfactory: one to five heterozygotes in F_1_ generation obtained with Cas9 protein injections compared to none or one when Cas9 mRNA was used (Table 4 and Supplementary Table 1).

**TABLE 4:** Comparison between the efficiency of Cas9 mRNA and Cas9 protein in generating heterozygotes in the targeted region of three genes *per* (short region – perS; perS 1 and perS 4 sgRNA were injected together), *pdf* (PDF 1) and *TEFLamide* (TEFL 2).

		**No of eggs**	**No of F_1_ heterozygotes/**
**Guide**	**Cas9 source**	**injected**	**No of G_0_ parents**
perS 1	mRNA (400 ng/μl)	145	0/0
perS 1	Protein (250 ng/μl)	180	0/0
perS 1	Protein (500 ng/μl)	165	2/1
perS 4	mRNA (400 ng/μl)	145	0/0
perS 4	Protein (250 ng/μl)	180	1/1
perS 4	Protein (500 ng/μl)	165	0/0
PDF 1	mRNA (400 ng/μl)	170	0/0
PDF 1	Protein (250 ng/μl)	179	1/1
PDF 1	Protein (500 ng/μl)	140	0/0
TEFL 2	mRNA (400 ng/μl)	158	0/0
TEFL 2	Protein (250 ng/μl)	151	0/0
TEFL 2	Protein (500 ng/μl)	120	2/2

### Guide RNA Source

**FIGURE 2:**
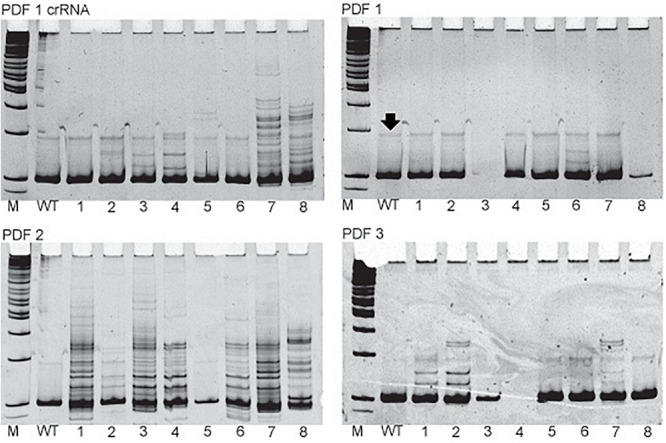
Examples of the *in vivo* sgRNA efficiency test. Pictures of polyacrylamide gels showing the heteroduplex mobility assay on eggs injected with different sgRNAs targeting *pdf* gene. The name of the particular sgRNA is mentioned above picture of the gel. M, marker; WT, wild type, single eggs screens are marked by numbers. Additional bands indicate high frequency of mosaicism in the egg (for example egg 1 in PDF 2 gel). Black arrow points to the nonspecific band occurring also in WT eggs and should not be mistaken for occurrence of mosaicism.

In all but one case, a 20-nt sequence of the crRNA was artificially fused with the tracrRNA, by the non-template PCR followed by *in vitro* transcription to synthesize chimeric sgRNA. In the case of producing *pdf* knockout mutant, commercially available crRNA was used for comparison of the efficiency (PDF 1 crRNA + tracrRNA). This crRNA targeted the same sequence as the PDF 1 chimeric guide RNAs used in above described trial. In this trial, different concentrations of Cas9 protein were also compared (Table 5). Our results show that commercially available sgRNA has higher efficiency (approximately 5 times) than chimeric sgRNA. Altogether five F_1_ heterozygotes were obtained using PDF 1 crRNA + tracrRNA, whereas only one was obtained by injection of the chimeric sgRNA PDF 1 (equal number of eggs were injected). Similar results were observed from later experiments testing the *in vivo* efficiency of different sgRNAs (Figure 2 and Supplementary Table 1), where the PDF 1 crRNA + tracrRNA injection resulted in 63.6% of eggs showing mosaicism compared to 5.2% mosaic eggs when injected with PDF 1 sgRNA. The higher concentration (18 μM) of PDF 1 crRNA + tracrRNA resulted in the production of three heterozygotes compared to two heterozygotes obtained by injection of 9 μM crRNA + tracrRNA.

**TABLE 5:** Comparison between the efficiency of the chimeric single guide RNA (sgRNA) produced by *in vitro* transcription and commercial crRNA targeting the same sequence in generation of heterozygotes in *pdf* gene.

			**No of eggs**	**No of F_1_ heterozygotes/**
**sgRNA**	**Cas9 source**	**guide source**	**injected**	**No of G_0_ parents**
PDF 1	Protein (250 ng/μl)	sgRNA (200 ng/μl)	179	1/1
PDF 1	Protein (500 ng/μl)	sgRNA (200 ng/μl)	140	0/0
PDF 1	Protein (250 ng/μl)	crRNA + tracrRNA (119 ng/μl + 200 ng/μl)	125	0/0
PDF 1	Protein (500 ng/m)	crRNA + tracrRNA (119 ng/μl + 200 ng/μl)	130	2/2
PDF 1	Protein (250 ng/μl)	crRNA + tracrRNA (238 ng/μl + 400 ng/μl)	106	0/0
PDF 1	Protein (500 ng/μl)	crRNA + tracrRNA (238 ng/μl + 400 ng/μl)	143	3/3

In this study, we compared the efficiency of the mutant production using the chimeric guide and crRNA only for one target sequence (*pdf* gene). In our hands, the use of the crRNA showed a higher mutation rate than chimeric sgRNA. These results, however, cannot possibly apply as a general rule. For instance, opposite results were reported in the fruit fly, *Ceratitis capitata* (Meccariello et al., 2017) and in case of zebrafish, the efficiency of the sgRNA and crRNA targeting the same region of the *tyrosinase* (*tyr*) and *spns2* gene was similar (Kotani et al., 2015). Due to the described in literature discrepancies concerning the efficiency of different sources of guide RNAs, the further trials of genome editing in *P. apterus* were done using chimeric sgRNA, whose cost of production and testing is considerably lower than the commercially available crRNAs.

### The Timing of Injections

In order to efficiently generate mutants, it is crucial to deliver CRISPR/Cas9 components in the right stage of embryonic development, when no cell membranes are formed. In most insects, early embryonic development follows a similar pattern. Immediately following fertilization the oocyte undergoes meiotic division (meiosis phase), and then the zygote nucleus undergoes several mitotic divisions within the central portion of the egg forming so-called “energids’ (cleavage division). Then nuclei migrate to the surface of the egg continuing their divisions without forming new cell membranes (syncytial blastoderm). Some nuclei migrate to the posterior part of the egg forming pole cells, the precursors of germ cells. Afterward cell membranes are formed, and the embryo reaches the stage of cellular blastoderm (Gilbert, 2000). Embryonic development of *P. apterus* takes around 1 week at 25°C. Early embryonic development, until the formation of the cellular blastoderm, takes around 1 day and consists of meiosis phase (0–4 h AEL), cleavage division (4–12 h AEL), and formation of the blastoderm (16–19 h AEL) (Socha, 1993).

Initially, *P. apterus* eggs were injected as early as possible (0–2 h AEL), in order to allow for Cas9 mRNA translation and action of the enzyme in the nuclei before cytokinesis. In this case, injections were administered into the middle of the egg, where the fertilized nuclei of the oocyte and later, energids should be present. However, because the cleavage division in the *P. apterus* eggs does not take place until the fourth-hour AEL, most probably only the oocyte or a small number of divided nuclei were present during injections, which decreases the chance of injecting sgRNA/Cas9 mixture in the proximity of any of the egg nuclei. For this reason, in order to increase the number of nuclei present during injections, eggs were injected in more advanced developmental time: 2–4 h AEL (eggs were collected every 2 h, and then injected 2 h afterward) or later 0–12 h AEL (eggs were collected after overnight egg laying and then injected immediately). In these cases, guide RNA and Cas9 protein mixture were injected into the posterior part of the egg, in the proximity of the future pole cells. Prolongation of the embryo development before injections did not change the rate of embryo survival (Table 6) when compared to injections performed in eggs 0–2 h AEL (*p* > 0.05, One Way ANOVA).

**TABLE 6:** Percentage of nymphs hatching after injections performed at different stages of egg development (combined results from several independent experiments – for details refer to Supplementary Table 1.

**Injections AEL (h)**	**Hatching (%)**
0–2	18.9–32.8
2–4	5.0–35.9
0–12	9.6–42.3

In order to compare the impact of the stage of *P. apterus* eggs development on the efficiency of the mutation rate, the mixture of the Cas9 protein and PDF 1 crRNA and tracrRNA, which gave the highest mutation rate in the eggs of 2–4 h AEL stage (Table 5), was injected into the eggs of 0–12 h AEL stage. Injections of eggs 2–4 h AEL resulted in production of 2 F_1_ heterozygotes, while injections of eggs 0–12 h AEL resulted in production of 21 F_1_ heterozygotes, even when significantly lower number of eggs were injected (Table 7). The results from this trial showed that the prolongation of the time of egg development before injection significantly increased the number of heterozygotes obtained in F_1_ generation (Table 7). Hence, all further sgRNA/Cas9 injections were performed on eggs at 0–12 h AEL stage.

**TABLE 7:** Comparison of number of heterozygotes found in F_1_ generation when young (2–4 h AEL) and mixed stages of eggs (0–12 h AEL) were injected with the identical CRISPR/Cas9 mixture targeting the same sequence of the *pdf* gene.

	**Injections**	**No of eggs**	**No of F_1_ heterozygotes/**
**Guide**	**AEL (h)**	**injected**	**No of G_0_ parents**
PDF1 crRNA + tracrRNA	2–4	130	2/2
PDF1 crRNA + tracrRNA	0–12	87	21/4

The timing of injections is crucial for successful mutant generation, and this step has to be optimized for any given species. Contrary to the studies reported in Lepidoptera (Li et al., 2015; Zhang and Reed, 2017) we found that injections performed in later embryonic stages give higher mutation rate when considering the number of heterozygotes in the F_1_ generation. However, we did not rigorously optimize this step further in order to pinpoint the optimal window for injections, as we already achieved a satisfactory increase in the efficiency of genome editing. Based on our experience we do not recommend injecting eggs at a very early stage of development if the species is known to have a prolonged early embryonic development, such as *P. apterus*. Nonetheless, our data and previous reports (Zhang and Reed, 2017) emphasize the significance of the optimization of the timing of egg injections for successful and efficient mutant production.

### sgRNA Efficiency Test

**FIGURE 3:**
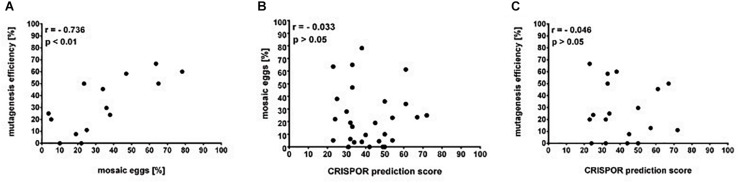
Guide RNAs on-target efficiency. **(A)** Mutagenesis efficiency (% of G_0_ parents producing F_1_ heterozygotes of the total fertile G_0_ parents) is positively correlated to the percentage of egg mosaics from the *in vivo* efficiency assay (Spearman correlation *p* < 0.01). **(B)** Percentage of eggs mosaicism and **(C)** mutagenesis efficiency is not correlated to the *in silico* prediction by the CRISPOR software (Spearman correlation *p* > 0.05).

The simple protocol was established to estimate the *in vivo* efficiency of the genomic DNA cleavage by the Cas9 combined with different sgRNAs. We designed and tested 29 sgRNAs targeting 5 genes (Table 1 and Supplementary Table 1). The eggs at 0–12 h AEL were injected with the sgRNA/Cas9 mixture (500 and 200 ng/μl respectively) and, after 30 h, embryonic DNA was isolated and subjected to the PCR with specific primers targeting the tested region. The heteroduplex mobility assay then tested the efficiency of the cleavage. The appearance of many additional bands, apart from the main PCR product, shows, that genomic DNA in embryo was cleaved and repaired by NHEJ mechanism. The frequency and intensity of additional bands in the heteroduplex mobility assay allow for an estimation of the level of mosaicism generated by particular sgRNA in the embryo (Figure 2). The most efficient sgRNAs (for example PDF 1 crRNA and PDF 2 showed in Figure 2) were then chosen for further mutant generation. Our *in vivo* eggs testing is a statistically significant predictor for the efficiency of the guide RNA in production of heritably modified adults (Spearman correlation *r* = 0.736, *p* < 0.01) (Supplementary Table 1 and Figure 3A). Therefore, for all further genome editing experiments, we used *in vivo* sgRNA efficiency test for choosing the appropriate guide for an efficient mutant generation. Similar evaluation method was used in the estimation of sgRNA and crRNA efficiency in zebrafish (Kotani et al., 2015). However, in that study, the efficiency was comparable between tested guide RNAs as well as the final mutant numbers.

### G_0_ Somatic and Germline Mosaicism Test

**FIGURE 4:**
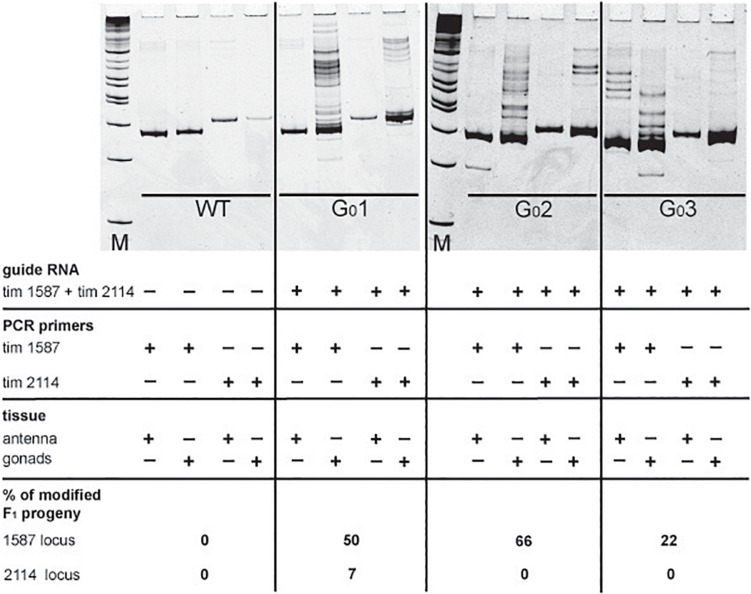
Images of polyacrylamide gels showing heteroduplex mobility assay performed on antenna and gonads of the wild type (WT) and G_0_ bugs (G_0_ bug 1–3). Eggs were injected simultaneously with the tim 1587 and tim 2114 sgRNAs. The same tissue was screened for mosaicism at the 1587 and 2114 loci. The lack of mosaicism in the somatic tissue (antenna) does not indicate the lack of gonadal mosaicism. The occurrence of gonadal mosaicism is a good predictor for the production of mutated progeny. Numbers below pictures indicate the percentage of mutated F_1_ progeny to the total progeny of the particular G_0_ parent (for detailed numbers see Supplementary Table 2).

In our initial attempts to generate mutants (N-terminal *cry2*, *per* short region, *TEFLamide* and *pdf* genes, Table 2 and Supplementary Table 1) all F_1_ progeny was screened (altogether, over 2600 F_1_) in order to find every existing heterozygote (12 heterozygotes were found). The screening techniques, both agarose and heteroduplex mobility assay are relatively cheap, but with the high number of individuals for screening, the cost of reagents and the labor needed for preparing the samples go up. It has to be taken into account that even when the sgRNA efficiency is high, not all of G_0_ founders will possess somatic or germline mosaicism, which could be transferred to the next generation. Therefore, by focusing only on the progeny of G_0_ mosaics, one can greatly reduce the number of individuals to be screened in order to isolate F_1_ individuals having mutation in the genes of interest. For a test of G_0_ founders mosaicism, two sgRNAs targeting the *timeless* gene (tim 1587 and tim 2114 showing different efficiency in embryonic sgRNA efficiency test 36 and 25%, respectively, Supplementary Table 1) were injected simultaneously along with the Cas9 protein. After adult eclosion, G_0_ bugs were allowed to mate with wild type bugs until the 1st instar of the F_1_ generation were produced. We screened for the level of the somatic mosaicism (antenna) and compared it to germline mosaicism (gonads) in the same G_0_ individuals, by the heteroduplex mobility assay. Figure 4 shows two representative gel images of the antenna vs. gonads screen mosaicism in WT and three different G_0_ bugs (G_0_1, G_0_ 2, and G_0_3). As shown in the Figure 4, the level of the somatic mosaicism is considerably lower than the mosaicism observed in gonads (compare lane 1 and 2 in G_0_ 1 bug). Additionally, neither tim 1587 nor tim 2114 sgRNA showed similar level of the somatic mosaicism to the level of mosaicism present in gonads (compare lane 1 and 2 in G_0_ 1 bug and lane 3 and 4 in G_0_ 2 bug) (Figure 4). Afterward, we analyzed for the number of F_1_ heterozygotes produced by a given individual. DNA was isolated from F_1_ adult’s antennae, and heteroduplex mobility assay was done separately for mutations occurring in the exon 7 (tim 1587 locus) and exon 8 (tim 2114 locus). All of the tested bugs had F_1_ progeny mutated in the loci 1587, and one of them, G_0_ 1 bug, also produced mutants with a mutation in 2114 loci. The numbers below gel images in Figure 4 indicate the % of heterozygous F_1_ individuals originating from the particular G_0_ parent.

It was proposed earlier that G_0_ mosaics can be selected by the screening of the cuticle appendage in Lepidoptera larvae (Markert et al., 2016). Based on this approach, most probably G_0_ bug #1 and G_0_ bug #2 (Figure 4), which showed no mark of somatic mosaicism in the antenna, would be discarded without further screening of their progeny. Those bugs, however, showed high level of mosaicism in gonads, and indeed produced several unique mutants. Our results indicate that somatic mosaicism found in antennae of G_0_ bugs cannot predict the level of germline mosaicism and that the level of gonadal mosaicism is a good prediction marker for the occurrence of hereditary mutations in the progeny. A similar finding was reported in a study performed on a larger scale to predict the level of germline mosaicism in zebrafish. The research showed that the level of somatic mosaicism found in screening G_0_ zebrafish fins is not a reliable marker for the number of genome modifications transferred to the F_1_ generation (Brocal et al., 2016).

According to these results, we started to screen only the F_1_ progeny of the G_0_ parents, which showed gonadal mosaicism (*pdf, per* SLIH region, *per* long region and *TEFLamide* genes). In some cases (tim 1587, tim 2114 perSLIH 3 and TEFL 5), not all G_0_ parents with gonadal mosaicism produced mutated progeny (Table 8). However, in most cases, all of the G_0_ parents produced at least one heterozygotic progeny. The efficiency of the transfer of the genome modifications to the F_1_ progeny varied between tested genes (ranging from 1.4 to 77.3% of mutated F_1_ bugs) and also between particular parents (Table 7 and Supplementary Table 3). On the other hand, when we screened the progeny of several G_0_ individuals, which did not show gonadal mosaicism, we never found any heterozygotes in F_1_ generation (for example per SLIH 1 in Supplementary Table 1).

**TABLE 8:** Percentage of gonadal mosaicism in G_0_ bugs injected with selected sgRNAs and the efficiency of the transfer of the genome modifications to the F_1_ progeny (for details refer to Supplementary Table 2).

	**No of G_0_**	**No of G_0_**	**No of G_0_ gonadal**	**% of F_1_**
	**fertile**	**gonadal**	**mosaics producing**	**heterozygotes**
**Guide**	**adults**	**mosaics (%)**	**F_1_ heterozygotes (%)**	**in F_1_ progeny**
tim 1587	48	23 (47.9)	16 (69.6)	4.2–66.7
tim 2114	48	11 (22.9)	6 (45.4)	5.0 – 26.7
C-cry2 194	14	5 (35.7)	5 (100)	1.4 – 7.4
perSLIH 3	10	2 (20.0)	0	–
perSLIH 4	4	2 (50.0)	2 (100)	8.3 – 22.5
perL 2	7	3 (42.8)	3 (100)	9.1 – 60.0
perL 3	12	7 (58.3)	7 (100)	5.0 – 61.5
PDF 1 crRNA	6	4 (66.6)	4 (100)	23.9 – 33.3
PDF 2	5	5 (100)	5 (100)	10.0 – 77.3
TEFL 3	9	6 (66.6)	6 (100)	9.1 – 35.7
TEFL 5	10	2 (20.0)	1 (50.0)	2.7

This way, by focusing on the progeny of G_0_ gonadal mosaics only, we significantly reduced the number of individuals screened in order to isolate progeny with mutations in the genes of interest. Therefore, we strongly suggest testing the level of mosaicism in gonads of the G_0_ individuals, not on the structures made entirely of somatic cells. This will help in screening only the progeny with the high probability of occurrence of mutations.

### *In silico* Guide Design and Efficiency Prediction

Many currently available online CRISPR design tools are reviewed in detail by Cui et al. (2018). Based on various algorithms and available genomic data, one can predict different sgRNAs, their on-target efficiency, and possible off-targets for multiple available Cas9 proteins. However, when working on non-model organisms, the complete genome of the species is rarely sequenced and available in public databases or, as in our case, the genomic assembly is still only partial. Available prediction tools for sgRNA are mostly focused on model organisms, and during CRISPR design one is forced to choose the genome of the particular species. One of the few tools which allow designing CRISPR experiments and predicting the on-target efficiency without the necessity of the information about the genome of the organism is CRISPOR^4^. It can find all available guide RNA target sites and predict their efficiency. Two different scores are available depending on the guide RNA delivery system (1) Fusi/Doench score for expression vector with U6 promoter or (2) Moreno-Mateos score for guide RNAs transcribed *in vitro* by the use of T7 promoter. Guide RNAs are scored in the range of values 0–100, where value 100 indicates the best target (Haeussler et al., 2016; Concordet and Haeussler, 2018). Here, we compared the predicted efficiency using the Moreno-Mateos score of all sgRNAs used in the study to the real data we obtained from the experiments (Table 9). We found no correlation between the CRISPOR predicted and observed *in vivo* efficiency in egg mosaicism or efficiency in production of heritable genetic modifications (Spearman correlation *p* = 0.86, *r* = −0.03 and *p* = 0.84, *r* = −0.04, respectively) Figures 3B,C. Others also observed a similar low correlation between *in silico* prediction and real sgRNA efficiency. It seems that not only the structure or composition of the guide RNA but also genomic features like DNA accessibility and compaction of the chromatin can also influence sgRNA efficiency (Modell et al., 2017; Labuhn et al., 2018; Lee et al., 2018). On the other hand, we found strong correlation between the efficiency of the sgRNAs tested *in vivo* (percentage of mosaics eggs) and percentage of G_0_ adults producing heterozygotes (Figure 3A, Spearman correlation *p* < 0.01, *r* = 0.74). In almost all cases, our *in vitro* efficiency test (except *perL* region) was a better predictor for the number of heterozygotes produced by the particular sgRNA (Table 9). However, also, in this case, the percentage of mosaic eggs observed after sgRNA injection is also not an absolute predictor of the sgRNA efficiency in the final number of mutants obtained. One has to remember that the efficiency of the germline mutations not only depends on the efficiency of the sgRNA used but also on the time of injections. Moreover, the fertilization of the mutated oocyte or fertilization by the mutated sperm is a random process and can affect the final number of mutated progeny. Still, the higher percentage of mosaicism observed in eggs leads to higher number of heterozygotes in the F_1_ generation (Figure 3A).

**TABLE 9:** sgRNA *in silico* and *in vivo* efficiency, compared to the final efficiency observed in the number of generated mutants.

	***In silico* efficiency**		**No of heterozygotes**
	**CRIPOR prediction**	***In vivo* efficiency**	**in F_1_ generation/**
**Guide**	**score**	**% of mosaic eggs**	**No of G_0_ parents**
N-cry2 2	44	^†^nd	0
N-cry2 3	32	^†^nd	0
N-cry2 8	57	^†^nd	7/6
C-cry2 188	38	4.0	^†^nd
C-cry2 194	25	38.0	11/5
C-cry2 196	30	28.0	^†^nd
tim 1448	61	61.3	^†^nd
tim 1481	54	23.1	^†^nd
tim 1517	47	4.5	^†^nd
tim 1587	50	36.0	64/16
tim 2114	72	25.0	14/6
tim 2193	46	0	^†^nd
perS 1	32	^†^nd	2/2
perS 4	52	^†^nd	1/1
perSLIH 1	24	22.0	0/0
perSLIH 2	40	9.5	^†^nd
perSLIH 3	50	10.0	0/0
perSLIH 4	67	23.5	19/2
perSLIH 5	54	5.2	^†^nd
perL 1	49	0	^†^nd
perL 2	33	65.0	30/3
perl 3	33	47.0	56/7
PDF 1 crRNA	23	63.6	21/4
PDF 1	23	5.2	1/1
PDF 2	38	78.2	80/5
PDF 3	32	19.2	^†^nd
TEFL 1	42	0	^†^nd
TEFL 2	34	3.7	2/2
TEFL 3	61	34	22/7
TEFL 4	31	0	^†^nd
TEFL 5	45	19	1/1
TEFL 7	32	6.2	^†^nd
TEFL 8	33	16	^†^nd
TEFL 9	50	0	^†^nd

**In vivo* efficiency column shows % of eggs displaying mosaicism when injected with particular sgRNA. The last column shows the % of heterozygotes of the total number of F_1_ individuals screened. •nd, not determined.*

### Off-Target Effects

As mentioned earlier, there are many available online CRISPR design tools for predicting off-targets of particular sgRNAs (Cui et al., 2018). However, in our case, where information on the complete genome is unknown, the prediction of off-target effects by any of the design tool is impossible. Even with the draft of the genome available, where the manual search of off-targets (search against the occurrence of the “seed” region of the crRNA (Cho et al., 2014; Zhang et al., 2015) can be performed, we cannot exclude the possibility that some of the off-targets exist in unassembled repetitive regions. Generally, off-target effects can result in lethality or loss of fertility when a developmentally important gene is affected. In our experiments, we noticed high mortality or lack of fecundity in both G_0_ and F_1_ heterozygotes in all of the genes tested (Supplementary Table 1 and Table 10) resulting in the low number of final mutant lines. Several factors could partially explain these low numbers. For example, the mortality of the G_0_ embryos is most probably the effect of injury during injections (compare numbers in Table 2 and Table 3). Even in the case of WT bugs, not all nymphs will mature into adults, and some adults will be infertile (in case of *P. apterus* the mortality of control nymphs is around 40–50% – and fertility of control females – 90–100%, data not shown). However, the low number of the established lines could also imply that, at least in some cases, this effect might be caused by the off-target gene mutations in genes essential for reproduction or development.

**TABLE 10:** Survival of lines derived from F_1_ heterozygotes.

	**No of F_1_**	**No of established**
**Guide**	**heterozygotes**	**lines**
N-cry2 8	6	5
C-cry2 194	11	3
tim 1587	64	5
tim 2114	14	0
perS 1	2	1
perS 4	1	0
perSLIH 4	19	7
perL 2	30	6
perL 3	56	28
PDF 1 crRNA	21	7
PDF 1	1	0
PDF 2	80	8
TEFL 2	2	0
TEFL 3	22	7
TEFL 5	1	0

Off-targets can also lead to inconclusive results when analyzing mutant phenotypes. The safest approach involves back-crossing all edited lines to the wild type bugs for several generations (we use 5–8 backcross generations) and create several independent mutants with different sgRNAs to exclude results obtained from off-target genome editing. Therefore, generation time of particular insect species is crucial for the whole experiment. Because the developmental cycle of the *P. apterus* takes around 30–40 days, the whole procedure of successful homozygous mutant line generation and reaching the number of insects in the stock that is sufficient for further experimental procedures can take approximately 1 year from the moment of the sgRNA design. This fact should be considered when introducing genome editing as a new tool in any laboratory, and a new insect species.

## Conclusion

Insects are remarkably diverse group inhabiting different ecological niches, with specialized adaptations to their habitat and performing complex behaviors. Some insects are also beneficial for human or agricultural pests. Therefore, functional research in non-model insect species beyond *Drosophila* is crucial to understand the mechanisms underpinning diverse processes and reveal its evolution. Carefully applied genome editing can be used for pest control. Until now, the research on the gene function in non-model insects mostly depended on external regulation of its expression by RNAi. It is known that the knock-down efficiency of RNAi is not always sufficient, and thus it may not be suitable for functional analysis of candidate genes in many insect species. The CRISPR/Cas9 technique allows us to surpass those problems by a generating mutants by a simple and relatively inexpensive method. However, usage of this technique is time-consuming and can present many unexpected problems. Our paper presents several optimizations steps which increased the efficiency of mutant generation significantly. The main steps that lowered the cost and labor needed in mutant production include the time of embryo injection, using heteroduplex mobility assay as a screening method, *in vivo* testing of sgRNA efficiency and G_0_ germline mosaicism screening. However, some of the optimization steps such as time of embryo injection or the method of egg injections are specific for the linden bug and needs to be optimized for other species. The other optimization steps can be used for any organism.

**FIGURE 5:**
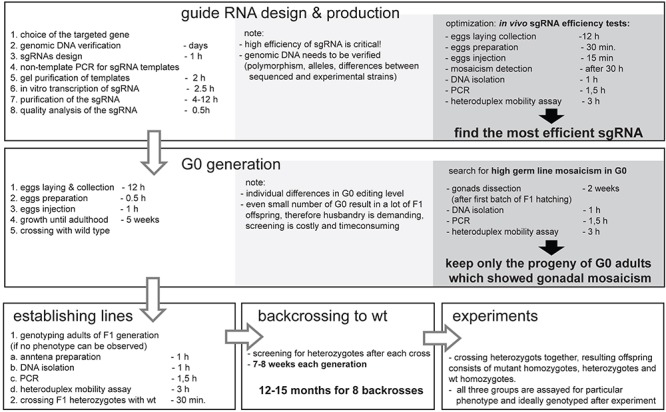
Simplified workflow of the CRISPR/Cas9 mutant production in non-model insect *Pyrrhocoris apterus* with key optimization steps, and indicated duration of experiments. Note that the duration depends on generation time of particular insect species, yet, the necessary backcrossing to wild type is the most time-consuming part of the entire experiment. The optimal time for injections is species specific, however, *in vivo* sgRNA efficiency test as well as detection of G_0_ gonadal mosaicism can be easily adapted to any insect species.

The development of the CRISPR/Cas9 genome editing allows for “fast” and “easy” mutant creation; nonetheless one need to consider the “hidden costs” before applying this technique to the project. Figure 5 represents the additional time and cost that needs to be considered when planning CRISPR/Cas9 genome editing experiments. The additional time needed involves several rounds of back-crosses, in order to avoid transmission of off-target mutation. The hidden cost is connected mainly with the materials and labor needed for mutants screening, which is multiplied by the number of genes targeted, different sgRNAs used, lines obtained, and the number of back-crossings performed. Despite these costs, this method is still affordable and can be applied in the research of the “non-model organism world.”

## Data Availability

All datasets for this study are included in the manuscript and/or the Supplementary Files.

## Author Contributions

DD and JK-R contributed to study design, interpretation of results, and wrote the manuscript. JK-R injected the embryos. JP assembled the draft of transcriptome and genome. LC, DC, LK, IF, MB, and BC-HW performed screenings. LC revised the manuscript. All authors gave final approval for publication and accept accountability for the content and work performed.

## Conflict of Interest Statement

The authors declare that the research was conducted in the absence of any commercial or financial relationships that could be construed as a potential conflict of interest.

## Footnotes

1 https://igtrcn.org

2 http://crispor.tefor.net

3 https://www.addgene.org/crispr/drosophila

4 http://crispor.tefor.net
