# Supplementary material for: CRISPR/Cas9 Genome Editing Introduction and Optimization in the Non-model Insect Pyrrhocoris apterus
Source: Front Physiol. 2019 Jul 15;10:891. doi: 10.3389/fphys.2019.00891 (PMC6644776; doi:10.3389/fphys.2019.00891)
Supplement: Supplementary file 2 [file Table_2.DOCX]

Supplementary Table 2. Examples of mutations generated in *P. apterus* by different guide RNAs. Mostly+/-10bp indels are found. Several mutants share the identical change in the target region.

| **Guide** | **Mutants sequences** |
| --- | --- |
| N-cry2 8 | WT CGCCTTCATGACAATCCAAGCCTTCGGCATGGTTTAAAAGGAGC  -5bp CGCCTTCATGACAATC-----CTTCGGCATGGTTTAAAAGGAGC /x2  +4bp CGCCTTCATGACAATCCA***T***G***A*CCGGA**TTCGGCATGGTTTAAAAGGAGC /x1  +13bp CGCCTTCATGACAAT***T*ATAAATGATGA**CAA***T***CC**AA**TTCGGCATGGTTTAAAAGGAGC /x1  +27bp CGCCTTCATGACAATCCA**AATGCCAGCAAAGGGGAGCTCCAAATA**CGCCTTCGGCATGGTTTAAAAGGAGC /x1 |
| C-cry2 194 | WT CTACTTACAGTTAATGAAATTCAGGCAGCCAGGTAATTTTTACTATACCA  -2bp CTACTTACAGTTAATGAAATTCAGGCAGCCAGG--ATTTTTACTATACCA /x2  -4bp CTACTTACAGTTAATGAAATTCAGGCAGCCAGG----TTTTACTATACCA /x1 |
| tim 1587 | WT AAGCAATCGATGAAACAATCTATAAAGAAGCAAGATGAAACACAGGTGAT  -16bp AAGCAATCGATGAAACAATCTATAAAGAA----------------GTGAT /x1  -11bp AAGCAATCGATGAAACAATCTATAAA-----------CATCACAGGTGAT /x2  -2bp AAGCAATCGATGAAACAATCTATAAAGAAGCAAGATGAA--ACAGGTGAT /x2 |
| perS1 | WT GATATAGTAACAGAGGAACAAAGCTTTTCGGTCAGTCTTGCTCCCACTTT  -2bp GATATAGTAACAGAGGAACAAAGCTT--CGGTCAGTCTTGCTCCCACTTT /x1 |
| perSLIH 4 | WT TTTTTTTTTGCAGTAGTGGGAGTTCGAAGTCCAGACAGAGCAAATCCAGTGGTGCACATGTCTCAGAACCA  -35bp TTTTTTTT-----------------------------------ATTATGTGGTGCACATGTCTCAGAACCA /x1  -6bp TTTTTTTTTGCAGTAGTGGGAGTTCGAAGTCCAGACAGAGCA------GTGGTGCACATGTCTCAGAACCA /x1  -5bp TTTTTTTTTGCAGTAGTGGGAGTTCGAAGTCCAGACAGAGCAAAT-----GGTGCACATGTCTCAGAACCA /x2  +13bp TTTTTTTTTGCAGTAGTGGGAGTTCGAAGTCCAGACAGAGCAAATCC**AGACAGAGCAAAT**AGTGGTGCACATGTCTCAGAACCA /x3 |
| perL 2 | WT GGAGCTTGGTGTAGTCGTTTCCATGGATGATGGCCTCGTAGTCTTTACAACTC  -4bp GGAGCTTGGTGTAGTCGTT----TGGATGATGGCCTCGTAGTCTTTACAACTC /x1  +2bp GGAGGAGCTTGGTGTAGTCGTTTCCATGG**TA**T*T*GATGGCCTCGTAGTCTTTACAACTC /x1  +4bp GGAGGAGCTTGGTGTAGTCGTTT**GTAG**CCATGGATGATGGCCTCGTAGTCTTTACAACTC /x1 |
| perL 3 | WT GGAGCTTGGTGTAGTCGTTTCCATGGATGATGGCCTCGTAGTCTTTACAACTC  -6bp GGAGCTTGGTGTAG------CCATGGATGATGGCCTCGTAGTCTTCACAACTC /x1  -6bp GGAGCTTGGTGTAGT-----CATGGATGATGGCCTCGTAGTCTTCACAACTC /x1  -6bp GGAGCTTGGTGTAGTCGT------GGATGATGGCCTCGTAGTCTTTACAACTC /x1  -5bp GGAGCTTGGTGTAGTCGT-----TGGATGATGGCCTCGTAGTCTTTACAACTC /x1  -4bp GGAGCTTGGTGTAGTCGCTT----GGATGATGGCCTCGTAGTCTTCACAACTC /x1  +1bp GGAGCTTGGTGTAGTCGTT**G***ATG*ATGGATGATGGCCTCGTAGTCTTTACAACTC /x1  +2bp GGAGCTTGGTGTAGTCGTTT**TG**CCATGGATGATGGCCTCGTAGTCTTTACAACTC /x1  +4bp GGAGCTTGGTGTAGTCGTTT**GTAG**CCATGGATGATGGCCTCGTAGTCTTTACAACTC /x2  +6bp GGAGCTTGGTGTAGTCGTT*G***GTGTAG**CCATGGATGATGGCCTCGTAGTCTTTACAACTC /x1  +6bp GGAGCTTGGTGTAGTCGT**GGTCGC**TTCCATGGATGATGGCCTCGTAGTCTTTACAACTC /x1  +10bp GGAGCTTGGTGTAGTCGTTT**GGATGATGGA**CCATGGATGATGGCCTCGTAGTCTTTACAACTC /x1 |
| PDF 1 crRNA | WT ACCTAGTAAAGGGCGAAGGACACGCACAAAAACGTAATTCGGAAATCATCAACTCACTACTCGGCATTCCAAAAGTTGTCAATGATG  -6bp ACCTAGTAAAGGGCGAAGGACACGCACAAAAA------TCGGAAATCATCAACTCACTACTCGGCATTCCAAAAGTTGTCAATGATG /x1  -5bp ACCTAGTAAAGGGCGAAGGACACGCACAAA-----AATTCGGAAATCATCAACTCACTACTCGGCATTCCAAAAGTTGTCAATGATG /x1  +6bp ACCTAGTAAAGGGCGAAGGACACGCACAAAAACGT**CGGAAA**AATTCGGAAATCATCAACTCACTACTCGGCATTCCAAAAGTTGTCA /x3 |
| PDF 2 | WT ACCTAGTAAAGGGCGAAGGACACGCACAAAAACGTAATTCGGAAATCATCAACTCACTACTCGGCATTCCAAAAGTTGTCAATGATG  -7bp ACCTAGTAAAGGGCGAAGGACACGCACAAAAACGTAATTCGGAAATCATCAACTC-------GGCATTCCAAAAGTTGTCAATGATG /x2  -4bp ACCTAGTAAAGGGCGAAGGACACGCACAAAAACGTAATTCGGAATCATCAACTCACT---C-GCATTCCAAAAGTTGTCAATGATG /x1  -3bp ACCTAGTAAAGGGCGAAGGACACGCACAAAAACGTAATTCGGAAATCATCAACTCACT---CGGCATTCCAAAAGTTGTCAATGATG /x4 |
| TEFL 3 | WT TAACATACAGTGTGGAAACGAAGAGTGCGGTTTGAAGAGGACCGTCGGAACTGAATTTCTTGGGAAAAGGGGAAGAGACTAAA  -32bp TAACATACAGTGTGGAAAC--------------------------------TGAATTTCTTGGGAAAAGGGGAAGAGACTAAA /x1  -10bp TAACATACAGTGTGGAAACGAAGAGTGCGGTTCGA-GAGG---------ACTGAATTTCTTGGGAAAAGGGGAAGAGACTAAA /x1  -8bp TAACATACAGTGTGGAAACGAAGAGTGCGGTTCGAAGAGGA--------ACTGAATTTCTTGGGAAAAGGGGAAGAGACTAAA /x4  +3bp TAACATACAGTGTGGAAACGAAGAGTGCGGTTTGAAGAGGAC*T*G**AAT**TCGGAACTGAATTTCTTGGGAAAAGGGGAAGAGACTAAA /x1 |

WT – wild type, ---- deletion, **insertion, s*ubstitution,*** PAM, /x number, number of lines with identical mutation
