# Supplementary material for: CRISPR/Cas9 Genome Editing Introduction and Optimization in the Non-model Insect Pyrrhocoris apterus
Source: Front Physiol. 2019 Jul 15;10:891. doi: 10.3389/fphys.2019.00891 (PMC6644776; doi:10.3389/fphys.2019.00891)
Supplement: Supplementary file 3 [file Table_3.DOCX]

Supplementary Table 3 Efficiency of the transfer of the genome modifications to the F_1_ progeny by the G_0_ gonadal mosaics.

| **Guide name** | **No of Go adults** | **No of Go gonadal mosaics** | **No of Go gonadal mosaics parents producing at least one F1 heterozygote** | **No of F1 heterozygotes produced by each individual Go mosaics parent** | **Total No of larvae produced by each individual Go mosaics parent** | **% of F1 hterozygotes to total No of F1 produced by each Go mosaics parent** |
| --- | --- | --- | --- | --- | --- | --- |
| **N-cry2 8** | 31 | 5 | 5 | 1 | 74 | 1.4 |
|  |  |  |  | 1 | 37 | 2.7 |
|  |  |  |  | 1 | 20 | 5.0 |
|  |  |  |  | 2 | 39 | 5.1 |
|  |  |  |  | 6 | 81 | 7.4 |
| **tim 1587** | 86 | 23 | 16 | 0 | 2 | 0.0 |
|  |  |  |  | 0 | 24 | 0.0 |
|  |  |  |  | 0 | 1 | 0.0 |
|  |  |  |  | 0 | 29 | 0.0 |
|  |  |  |  | 0 | 5 | 0.0 |
|  |  |  |  | 0 | 10 | 0.0 |
|  |  |  |  | 0 | 17 | 0.0 |
|  |  |  |  | 1 | 24 | 4.2 |
|  |  |  |  | 1 | 20 | 5.0 |
|  |  |  |  | 1 | 17 | 5.9 |
|  |  |  |  | 1 | 11 | 9.1 |
|  |  |  |  | 1 | 8 | 12.5 |
|  |  |  |  | 2 | 15 | 13.3 |
|  |  |  |  | 4 | 28 | 14.3 |
|  |  |  |  | 2 | 11 | 18.2 |
|  |  |  |  | 4 | 19 | 21.1 |
|  |  |  |  | 7 | 32 | 21.9 |
|  |  |  |  | 7 | 30 | 23.3 |
|  |  |  |  | 4 | 14 | 28.6 |
|  |  |  |  | 7 | 14 | 50.0 |
|  |  |  |  | 17 | 30 | 56.7 |
|  |  |  |  | 2 | 3 | 66.7 |
|  |  |  |  | 2 | 3 | 66.7 |
| **tim 2114** | 86 | 11 | 6 | 0 | 11 | 0.0 |
|  |  |  |  | 0 | 19 | 0.0 |
|  |  |  |  | 0 | 3 | 0.0 |
|  |  |  |  | 0 | 17 | 0.0 |
|  |  |  |  | 0 | 8 | 0.0 |
|  |  |  |  | 1 | 20 | 5.0 |
|  |  |  |  | 1 | 15 | 6.7 |
|  |  |  |  | 1 | 14 | 7.1 |
|  |  |  |  | 1 | 14 | 7.1 |
|  |  |  |  | 2 | 24 | 8.3 |
|  |  |  |  | 4 | 15 | 26.7 |
| **perSLIH 3** | 18 | 2 | 0 | 0 | 3 | 0.0 |
|  |  |  |  | 0 | 101 | 0.0 |
| **perSLIH 4** | 9 | 2 | 2 | 3 | 36 | 8.3 |
|  |  |  |  | 16 | 71 | 22.5 |
| **perL 2** | 8 | 3 | 3 | 4 | 44 | 9.1 |
|  |  |  |  | 16 | 37 | 43.2 |
|  |  |  |  | 9 | 15 | 60.0 |
| **perL 3** | 21 | 7 | 7 | 2 | 40 | 5.0 |
|  |  |  |  | 5 | 33 | 15.2 |
|  |  |  |  | 8 | 38 | 21.1 |
|  |  |  |  | 4 | 15 | 26.7 |
|  |  |  |  | 21 | 44 | 47.7 |
|  |  |  |  | 7 | 14 | 50.0 |
|  |  |  |  | 8 | 13 | 61.5 |
| **PDF 1 crRNA** | 10 | 4 | 4 | 11 | 46 | 23.9 |
|  |  |  |  | 9 | 37 | 24.3 |
|  |  |  |  | 21 | 86 | 24.4 |
|  |  |  |  | 1 | 3 | 33.3 |
| **PDF 2** | 7 | 5 | 5 | 1 | 10 | 10.0 |
|  |  |  |  | 2 | 7 | 28.6 |
|  |  |  |  | 15 | 40 | 37.5 |
|  |  |  |  | 45 | 66 | 68.2 |
|  |  |  |  | 17 | 22 | 77.3 |
| **TEFL 3** | 16 | 5 | 5 | 2 | 22 | 9.1 |
|  |  |  |  | 8 | 57 | 14.0 |
|  |  |  |  | 4 | 26 | 15.4 |
|  |  |  |  | 3 | 12 | 25.0 |
|  |  |  |  | 5 | 14 | 35.7 |
| **TEFL 5** | 18 | 2 | 2 | 0 | 10 | 0.0 |
|  |  |  |  | 1 | 37 | 2.7 |
