# Supplementary material for: CRISPR/Cas9 Genome Editing Introduction and Optimization in the Non-model Insect Pyrrhocoris apterus
Source: Front Physiol. 2019 Jul 15;10:891. doi: 10.3389/fphys.2019.00891 (PMC6644776; doi:10.3389/fphys.2019.00891)
Supplement: Supplementary file 4 [file Data_Sheet_1.PDF]

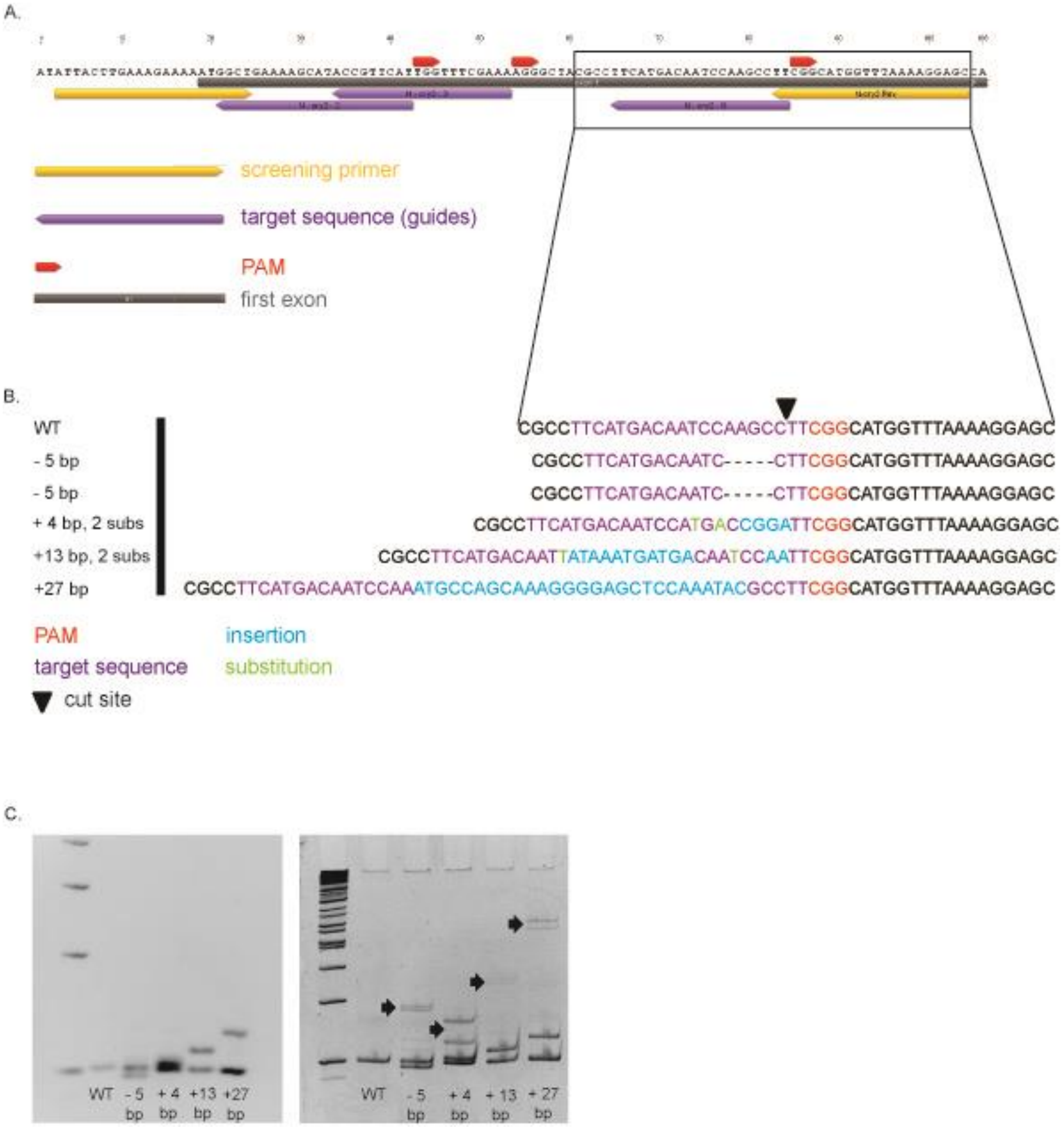

Supplementary Figure 1 A) Graphical illustration of the genomic fragment of of the *P. apterus cryptochrome2* gene with the part of the first exon marked. 3 different sgRNAs (violet) were designed and used for mutants production. Only sgRNA N-cry2-8 resulted in production of mutated heterozygotes. Out of over 2000 F1 heterozygotes screened only 6 individuals showed mutation in the target region (Suppl. Table 1) B) Sequences of the mutations found in established lines of the *P. apterus cry2* mutants. Errors in NHEJ resulted in deletions, insertions and substitutions in the targeted region. C) Comparison of the resolution of 4% agarose gel (on the left) and PAGE heterodplex mobility assay (on the right). The same samples of *cry2* heterozygote mutants were run simultaneously on both gels. WT – wild type, - and + indicates deletion and insertion, respectively. Arrows on the picture of the gel indicate additional bands - a marker of formation of heteroduplexes of the WT and mutated DNA in heterozygotes.
